# Phylogenetic patterns and conservation among North American members of the genus *Agalinis *(Orobanchaceae)

**DOI:** 10.1186/1471-2148-8-264

**Published:** 2008-09-26

**Authors:** James B Pettengill, Maile C Neel

**Affiliations:** 1 Behavior, Ecology, Evolution, and Systematics Graduate Program, University of Maryland College Park, College Park, MD 20742 USA; 2 Department of Plant Science and Landscape Architecture and Department of Entomology, University of Maryland College Park, College Park, MD 20742 USA

## Abstract

**Background:**

North American *Agalinis *Raf. species represent a taxonomically challenging group and there have been extensive historical revisions at the species, section, and subsection levels of classification. The genus contains many rare species, including the federally listed endangered species *Agalinis acuta*. In addition to evaluating the degree to which historical classifications at the section and subsection levels are supported by molecular data sampled from 79 individuals representing 29 *Agalinis *species, we assessed the monophyly of 27 species by sampling multiple individuals representing different populations of those species. Twenty-one of these species are of conservation concern in at least some part of their range.

**Results:**

Phylogenetic relationships estimated using maximum likelihood analyses of seven chloroplast DNA loci (aligned length = 11 076 base pairs (bp) and the nuclear ribosomal DNA ITS (internal transcribed spacer) locus (733 bp); indicated no support for the historically recognized sections except for Section Erectae. Our results suggest that North American members of the genus comprise six major lineages, however we were not able to resolve branching order among many of these lineages. Monophyly of 24 of the 29 sampled species was supported based on significant branch lengths of and high bootstrap support for subtending branches. However, there was no statistical support for the monophyly of *A. acuta *with respect to *Agalinis tenella *and *Agalinis decemloba*. Although most species were supported, deeper relationships among many species remain ambiguous.

**Conclusion:**

The North American *Agalinis *species sampled form a well supported, monophyletic group within the family Orobanchaceae relative to the outgroups sampled. Most hypotheses regarding section- and subsection-level relationships based on morphology were not supported and taxonomic revisions are warranted. Lack of support for monophyly of *Agalinis acuta *leaves the important question regarding its taxonomic status unanswered. Lack of resolution is potentially due to incomplete lineage sorting of ancestral polymorphisms among recently diverged species; however the gene regions examined did distinguish among almost all other species in the genus. Due to the important policy implications of this finding we are further evaluating the evolutionary distinctiveness of *A. acuta *using morphological data and loci with higher mutation rates.

## Background

The increase in use of molecular systematics in studies of angiosperm evolution has resulted in numerous phylogenies describing relationships across a range of evolutionary history [1]. Studies of closely related species [2] are particularly important for filling in the tips on the angiosperm tree of life [3]. Phylogenetic hypotheses of the evolutionary relationships among members of the same genus provide frameworks for comparative research on mechanisms of diversification and speciation [4]. These phylogenies are also valuable resources for people concerned with conservation in that they provide a relatively objective means of quantifying evolutionary distinctiveness and resolving taxonomic ambiguities involving rare taxa [5-8]. It is this application to identifying lineages that are sufficiently distinct to warrant taxonomic status and thus are eligible for legal protection (i.e., species, subspecies, and varieties) that greatly interests us.

A basic assumption of many species concepts [e.g. [9-13]] and operational species delimitation methods [14] is that individuals of one species share common ancestry to the exclusion of members of other species. This shared common ancestry, which is a logical consequence of reproductive isolation between two groups, is expected to ultimately be reflected by genealogical exclusivity or monophyletic relationships inferred from phylogenetic analysis of DNA sequence or fragment data [11]. However, the rapidly accumulating phylogenies of congeneric taxa with mismatches between gene trees and an expected species tree [15,16] is yielding a startling picture of the extent to which the expectation of monophyly is not met. Such mismatches can indicate imperfect taxonomy, lack of sufficient variation to detect differentiation, incomplete lineage sorting of shared ancestral polymorphisms, or contemporary hybridization or introgression. The amount of evolutionary time required for mutations to accumulate and for shared ancestral polymorphisms to sort out after speciation events [17-19] can make distinguishing among recently diverged taxa quite challenging. Coalescent theory predicts that it will take on the order of ~8.7 N_e _generations for reciprocal monophyly of neutral, biparentally inherited loci to evolve in diverging lineages [17,20]. Thus, although the degree and duration of isolation necessary to achieve monophyly (especially across multiple loci) guarantees the evolutionary independence of monophyletic operational taxonomic units, absence of evidence for such independence, however, cannot automatically be assumed to mean that two entities are not reproductively isolated [21]. In these cases, additional evidence will be required to resolve ambiguities.

In this study, we examined phylogenetic relationships among 29 North American *Agalinis *(Raf.) species. This genus of flowering plants is restricted to the Western Hemisphere where approximately 40 species occur in the eastern and central United States and Canada and approximately 30 species are found in South America, Mexico, and Central America [22-27]. Due to taxonomic uncertainties, the exact number of species in the genus is unclear; acceptance of particular taxa varies across authors and taxonomic revision is in progress. Historically, *Agalinis *was considered to be part of the family Scrophulariaceae but multiple phylogenetic analyses support placement in the family Orobanchaceae [28-30]. This plant family was traditionally associated with holoparasitism; however the broadened concept includes a number of autotrophic genera, such as *Agalinis*, that are hemiparasitic.

The majority of North American *Agalinis *species grow on the coastal plains of southern and southeastern North America. A secondary concentration of species occurs in the midwestern part of the continent and an even smaller number of species extend to the piedmont and to the coastal plains of the Mid-Atlantic, New England and the southern reaches of the Maritime Provinces in Canada [22-24,31,32]. Throughout this geographic range, habitats occupied by *Agalinis *species are typically grasslands and savannas, grassy openings in woodlands and forests, or other herb dominated habitats. Soil moisture requirements vary greatly across taxa, ranging from inundated wetlands (including bogs, streams, ponds, and salt marshes), to wetland edges, to dry uplands. Because *Agalinis *species thrive in relatively open sites with no or low cover of shrubs and trees, many of them are found in early successional habitats and are most abundant following fire or other disturbance events. Due to overall declining trends in grassland extent and condition resulting from both development and lack of natural disturbance, a number of *Agalinis *species are increasingly restricted to forest edges and anthropogenically maintained openings such as utility corridors, and road verges. Although the more ruderal species can be extremely abundant and widespread in these highly modified habitats, our observation indicated that some less abundant and more geographically restricted species are susceptible to mowing during the reproductive season, insufficient disturbance to remove woody vegetation, herbicide applications, and invasions of aggressively competitive non-native species.

General characteristics of the genus *Agalinis *include membranaceous, ephemeral corollas mostly with red-purple spots and yellow guide lines and wingless seeds that have variously reticulate seed coats [22,23,33,34]. Beyond the above characteristics, life form, morphology, anatomy, and floral form and color are variable, particularly in South American taxa. Unfortunately, relationships among the South American taxa are poorly understood, they are not included in any published classification schemes for the genus, and we were unable to obtain material to include them in this work. With exception of the perennial species *A. linifolia*, all North American species are annual herbs and all but three species (*A. auriculata*, *A. densiflora*, and *A. heterophylla*) have linear to filiform or scale-like leaves. Although mating systems have not been described for all members of the genus, the species that have been investigated include obligate outcrossing (*A. strictifolia *[35]), mixed mating (*A. acuta *[36], *A. skinneriana *[37], *A. obtusifolia *[38], *A. decemloba *[38], and *A. auriculata *[39]), and predominantly selfing due to cleistogamy (*A. neoscotica *[40]).

The genus is taxonomically difficult and there have been numerous revisions of species and subspecies. In addition to taxonomic uncertainties, relationships among *Agalinis *species have been enigmatic and section-level classifications have been anything but stable. Pennell [23] originally suggested five sections within the genus but later suggested only three sections with five subsections [22]. Work based on seed, stem and leaf, and seedling characteristics as well as karyotypes [34,41-46] yielded revisions to Pennell's classification that recognized five sections (Erectae, Heterophyllae, Linifolieae, Purpureae, and Tenuifolieae) and three subsections within the Purpureae (Pedunculares, Purpureae, and Setaceae). Previous phylogenetic analysis of 15 *Agalinis *species based on 7323 aligned bp of nucleotide sequence variation at three cpDNA loci (*rbc*L, *ndh*F, and *mat*K) [47] did not fully support either Pennell's or Canne-Hilliker's section-level classifications, although one section and some subsections suggested by Canne-Hilliker appeared to represent natural groups. Specifically, monophyly of Section Erectae was supported but Sections Purpureae and Heterophyllae were polyphyletic. Subsection Pedunculares was monophyletic but did not appear to be related to other Section Purpureae taxa as had been presumed. Limited taxon sampling and relatively low cpDNA sequence variation in that study prevented more thorough evaluation of relationships among Sections Linifoliae and Tenuifolieae and other subsections within the Purpureae.

In the present study, we provide a more comprehensive phylogenetic treatment of the genus by examining 29 North American *Agalinis *species using 7 cpDNA loci and 1 nuclear locus. Our specific objectives included simultaneously evaluating the monophyly of sections, subsections, and species that have been named solely based on anatomy and morphology. Every polytypic section and subsection is represented by multiple species and 27 species are represented by multiple individuals. In contrast to traditional sampling approaches in systematics studies that include only one representative per species [15,48], we were able to treat species labels as testable hypotheses [49]. The extensive sampling also provides a genus-wide context in which to evaluate the amounts and patterns of divergence among putative species that can be detected using the loci we sampled. This context is particularly critical for interpreting cases in which we fail to detect differentiation.

In addition to describing the evolutionary relationships among the sampled individuals, this study has important implications for conservation. We sampled 21 species that are considered imperiled (S2) or critically imperiled (S1) in at least 1 state in which they occur; 6 of these species are also globally vulnerable (G3 or G3–G4) and 3 are critically imperiled (G1) [Table 1; [50]]. Data on the divergence of and relationships among such a large number of species of conservation concern can help prioritize rare species for conservation [51,52] by estimating their degree of uniqueness within the genus. We were specifically interested in addressing questions regarding the taxonomic status of three sets of species whose distinctiveness from one another and thus conservation status had previously been questioned: *A. acuta *and *A. tenella*, *A. tenella *and *A. obtusifolia*, *A. decemloba *and *A. obtusifolia*. The status of *A. acuta *has been questioned previously and Neel and Cummings [47] found only a single nucleotide difference between *A. acuta *and *A. tenella *across 4048 bp of cpDNA that included *rbc*L and *mat*K. *Agalinis acuta *occurs on the coastal plain in eastern Massachusetts; Rhode Island; on Long Island, New York; and in Maryland. *Agalinis tenella *occurs on the coastal plain from South Carolina south to Florida and west to Alabama [22]. Morphologically, *A. acuta *is distinguished from *A. tenella *by having a shorter corolla, smaller seeds, and shorter pedicels [22]. We were interested in the other two sets of species because the current taxonomic treatment in the USDA PLANTS database [27] suggests that *A. tenella *and *A. decemloba *are synonymous with *A. obtusifolia *[53]. If this taxonomic treatment is accurate and *A. acuta *is also not distinguishable, combining all four taxa would be appropriate and there would be important conservation policy consequences. As originally described, *A. decemloba *grows on the piedmont in Virginia, North Carolina, and South Carolina [22,23]. *Agalinis obtusifolia *is known from collections from Maryland south to Florida and then west through Georgia to Louisiana on both the piedmont and the coastal plain. Clarifying the taxonomic status of *A. acuta *[54] is essential because if it is not a species, subspecies, or variety it is not eligible for listing under the U.S. Endangered Species Act [55]. If it is synonymous with other species, the status of *A. acuta *would need to be revised based on the distribution, abundances, and threats of the populations representing those other species.

**Table 1:** North American *Agalinis *species including the number of individuals (N) and conservation status of all species included in this study.

Taxon^1^	N^2^	Status^3^
**Section Erectae ***(n = 13)*		
*A. acuta*	9	G1/S1
*A. aphylla*	2	G3–G4/S2
*A. decemloba*	2	NR
*A. gattingeri*	3	G4/S1
*A. obtusifolia*	5	G4–G5-Q/S1
*A. oligophylla*	3	G4/S1
*A. skinneriana*	3	G3/S1
*A. tenella*	6	NR
*A. viridis*	2	G4/S1
*A. keyensis*	NS	
**Section Heterophyllae ***(n = 14)*		
*A. auriculata*	2	G3–G4/S1
*A. calycina*	1	G1/S1
*A. heterophylla*	3	G4–G5/S1
*A. densiflora*	NS	
**Section Linifoliae***(n = 14)*		
*A. linifolia*	2	G4?/S1
**Section Purpureae**		
Subsection Pedunculares *(n = 13)*		
*A. edwardsiana*	1	G4/S4
*A. homalantha*	2	G5/S1
*A. pulchella*	2	G4–G5/S3?
*A. strictifolia*	2	G4/SNR
*A. navasotensis*	2	G1/S1
*A. peduncularis*	NS	
*A. aspera*	NS	
Subsection Purpureae *(n = 14)*		
*A. fasciculata*	3	G5/S1
*A. harperi*	2	G4?/SNR
*A. maritima*	2	G5/S2
*A. paupercula*	2	G5/S1
*A. purpurea*	4	G5/S1
*A. tenuifolia*	3	G5/S1
*A. pinetorum*	NS	
*A. neoscotica*	NS	
*A. virgata*	NS	
Subsection Setaceae *(n = 14)*		
*A. laxa*	2	G3–G4/S3?
*A. plukenettii*	2	G3–G5/S1
*A. setacea*	2	G5?
*A. stenophylla*	NS	
*A. filifolia*	NS	
**Section Tenuifolieae ***(n = 14)*		
*A. filicaulis*	2	G3–G4/S1
*A. divaricata*	2	G3?/S1
*A. nutallii*	NS	
**Outgroup Species**		
*Aureolaria pectinata*	1	G5?
*Aureolaria pedicularia*	1	G5
*Brachystigma wrightii*	1	G4
*Dasistoma macrophylla*	1	G4

^1^Chromosome counts represent those known for the section or subsection based on extensive species sampling [43,45].

^2^NS = Not Sampled.

^3^Conservation Status: global ranking (G1 = critically imperiled; G2 = imperiled; G3 = vulnerable to extinction or extirpation; G4 = apparently secure; G5 = demonstrably secure or widespread)/highest state ranking for each species (S1–S5 are equivalent to the global scale but applied to within a single state) (USA); when a range or question mark (?) is given the precise conservation status is uncertain.; NR and SNR = not ranked.

## Methods

### Taxon sampling

A total of 79 individuals representing 29 out of the ~40 putative North American *Agalinis *species were included in this study (Table 1). The sampled species represented all North American sections and subsections and all polytypic groups were represented by more than one species. The number of individuals per species ranged from 1–9 and when multiple individuals were used, they were from different populations. Sample locations for most species were selected somewhat opportunistically and often coincided with locations sampled for anatomical and morphological work by Dr. J. Canne-Hilliker. We attempted to include samples from geographically distinct locations for each species in order to capture the potential range of within-species variation (Additional file 1). Samples of *A. acuta *represent all geographic regions from which this species is known, and include most extant populations (Additional file 1). Samples of *A. obtusifolia *and *A. tenella *were also distributed to represent the range of each species (Additional file 1). The two *A. decemloba *populations were from the north central portion of the range. One representative of each of four outgroup species was also sampled: *Aureolaria pedicularia *(L) Raf., *Aureolaria pectinata *(L) Raf., *Brachystigma wrightii *(A. Gray) Pennell, and *Dasistoma macrophylla *(Nutt.) Raf. Fifteen of the *Agalinis *individuals and three of the four outgroup individuals were the same as those used in the previous phylogenetic study of the genus and related genera (*Aureolaria pectinata *is new and *Seymeria pectinata *Pursh was not included) [47]. Vouchers are located at University of Guelph, University of Maryland, Iowa State University, and University of Texas Austin. Specific information on the location of particular specimens is available on request. We did not collect voucher specimens from the endangered *A. acuta *because these populations are well documented by state Natural Heritage Programs and the U.S. Fish and Wildlife Service.

### DNA isolation, amplification, and sequencing

Total genomic DNA was isolated from fresh or frozen (-80°C) leaves and flower buds by grinding 50–100 mg of tissue to powder in liquid nitrogen with a mortar and pestle, and then using GenElute Plant Genomic DNA Kits (Sigma Chemical Company, St. Louis, Missouri, USA) or Qiagen DNEasy Kits (Qiagen Corporation, Valencia, California USA) following manufacturer's instructions.

We analyzed sequences from seven chloroplast gene regions (*mat*K, *rbc*L, *ndh*F, *trn*T (UGU)-*trn*F (GAA), *rps*2, *rpo*B, and *psb*A-*trn*H) and the nuclear DNA (nDNA) locus ITS (18S-5.8S-26S). The first three cpDNA loci were used in the previously mentioned study [47] and they represent relatively slowly evolving portions of the chloroplast genome. Although there is rate variation among sites within these loci [47] that inform different levels of the phylogeny, they are most useful for resolving more ancestral relationships. The other chloroplast loci and the nuclear ITS locus were chosen because they have been shown to be informative at distinguishing among recently diverged taxa and even among populations within species [56-58]. Our strategy was to assay *rbc*L and *mat*K from at least one individual of each species to resolve the deeper relationships within the genus. We then attempted to sequence the other five loci from all sampled individuals. All but two of these loci were amplified using a single forward and reverse primer pair. The exceptions were *trn*T-*trn*F which required two PCR reactions per individual using *trn*T-a/*trn*L-d and *trn*L-c/*trn*F-f [59]. The *rps*2[28] locus was problematic for certain species but amplifications using the alternative forward primer *rps*2-47F, instead of *rps*2-18F, were successful. Details of amplification and sequencing for *rbc*L and *mat*K are given in Neel and Cummings [47]. In previous work *ndh*F was extremely difficult to amplify from a number of *Agalinis *species and although we did not pursue additional *ndh*F sequences, we used the ones available from Neel and Cummings [47] in our analysis.

Despite the well documented problems with using ITS for phylogenetic analyses, due to high copy number and difficulty optimizing PCR [e.g., [60]], we reliably obtained sequences using two primer pairs (ITS4 and ITS5 or ITS1 and ITS4)[61]. These primers did yield multiple PCR products and attempts to design species-specific primers for these taxa did not sufficiently reduce the number of copies. We therefore extracted the desired PCR product, (identified as the brightest band nearest to the target size), from an agarose gel using Qiagen's QIAquick Gel Extraction Kit according to the manufacturer's protocol. Inspection of the sequence trace curves confirmed that only a single copy had been sequenced.

All polymerase chain reactions (PCR) were done with Eppendorf MasterTaq PCR kits (Brinkman, Westbury, New York, USA) on MJ Research PTC-200 Thermal Cyclers. In general, the PCR temperature profile was 30 cycles of 94°C for 60 s, annealing temperature set approximately 5°C below the lower of the two primer melting temperatures for 90 s, 72°C for 150 s, and a final 15 min elongation period at 72°C. Amplified DNA fragments were purified using the Qiagen QIAQuick PCR Purification Kit according to manufacturer's instructions, unless noted otherwise.

Because many of the individuals and species we investigated were closely related and thus sequence variation was likely to be low, four replicate sequencing reactions were carried out for both forward and reverse primers for a given locus, resulting in eight-fold coverage across most regions of all loci. This conservative sequencing strategy ensured accuracy and prevented erroneous base calls associated with sequencing error that can cause serious issues when only single sequences are analyzed. Sequencing reactions were conducted with BigDye Terminator v3.1 Cycle Sequencing chemistry (Applied Biosystems, Foster City, California, USA) with reactions set up in 96-well microtiter plates. Total reaction volume was 7 μl (1–3 μl DNA template, 1.5 μl 5× Sequencing Buffer, 1 μl primer [25 μM], 0.5 μl BigDye Terminator, and 1–3 μl ddH2O). Cycle sequencing of purified PCR product was performed on an MJ Research PTC-200 Thermal Cycler and subsequent cleanup and preparation for sequencing was performed according to the manufacturer's protocol.

### Data analysis

The program Sequencher v4.6 (Gene Codes Corporation, Ann Arbor, Michigan, USA) was used for base calling, quality assignments, and assembling consensus sequences for each sample from the replicate bi-directional sequence reads. Contigs for each locus exported from Sequencher were aligned using the default settings of MUSCLE [62]. BioEdit [63] was used to manually edit alignments of the cpDNA loci *rps*2, *trn*T-*trn*F, and *psb*A-*trn*H which had numerous insertions/deletions. Alignments were exported as FASTA files and then converted to non-interleaved NEXUS files using MacClade v4.06 [64]. Three different data matrices were created: 1) cpDNA only, 2) nuclear ITS sequences only, and 3) a concatenation of all sequences.

To evaluate the variability of each locus, we calculated the number of characters that were constant, parsimony informative, and autapomorphic using the default parsimony settings in PAUP* [65]. We also estimated the maximum likelihood pairwise distances between sampled individuals within and among *Agalinis *species for each locus separately. Nucleotide substitution model parameters for the maximum likelihood distance measures were chosen using MODELTEST [66]. MODELTEST evaluates the likelihood scores of the same neighbor-joining tree for each of the 56 nucleotide substitution models calculated using PAUP* and the best fitting model was chosen using Akaike's Information Criterion (AIC).

Phylogenetic analyses were performed using the program GARLI v0.951 (Genetic Algorithm for Rapid Likelihood Inference) [67]. GARLI performs heuristic phylogenetic searches under the GTR + Γ + I (General Time Reversible with Gamma distributed rate heterogeneity and a proportion of invariant sites [68,69]) nucleotide substitution model where topologies are evaluated based on their likelihood. The program calculates the maximum likelihood of a topology in the same manner as PAUP* but uses a genetic algorithm [70] to more efficiently evaluate alternative topologies. For each dataset, the best tree was found by running GARLI on the original data matrix with the default settings. We used likelihood ratio tests as implemented in PAUP* to assess whether branch lengths associated with the best topology inferred with GARLI were significantly greater than zero. To estimate the support for each node, phylogenies were created for 1000 bootstrap replicates of each dataset. A 50% majority rule consensus tree of the 1000 bootstrap replicates from GARLI was then created using PAUP*. The support values at each node on the consensus tree were added to the best tree found by GARLI, which allowed us to display both node support values and branch lengths.

To decrease the computational time required to complete the bootstrap replicates we reduced the number of generations that were performed without finding a better scoring topology before a replicate was terminated from 10 000 to 5000. To complete the bootstrap analyses for the cpDNA and all loci combined datasets in a relatively short time we used Grid computing through The Lattice Project [71]. The GARLI executable was converted to a Grid service such that batches of bootstrap replicates were distributed among hundreds of computers where they were conducted asynchronously in parallel [72]. The 1000 bootstrap replicates for the smaller ITS dataset were accomplished on a single desktop computer.

We used the approximately unbiased (AU) test [73] as implemented in the program CONSEL [74] to evaluate whether a tree that constrained both *A. acuta *and *A. tenella *to be monophyletic was significantly worse than the best tree from an unconstrained analysis using the same data set. We repeated this test for each of the three data matrices. The AU test calculates a probability value of different topologies from bootstrap replicates of the site-likelihoods [73]. We also used the AU test to determine the influence of missing data on phylogenies inferred from the cpDNA and ITS datasets and to assess the degree of congruence between the phylogeny based on cpDNA loci and the phylogeny based on the complete data set. It is not possible to directly assess the incongruence between the topologies from the concatenated cpDNA dataset and the ITS locus because the data matrices differed in the number of individuals. However, given that the cpDNA dataset and the all-loci-combined dataset differed only in the inclusion of ITS, we used the AU test to compare these two topologies as a means to estimate the incongruence with the cpDNA phylogeny introduced by the ITS locus.

## Results and Discussion

### Characteristics of the sampled loci

Despite extensive efforts, it was not possible to obtain sequences of all loci for all species (Table 2 & Additional file 1). Total aligned length of the cpDNA dataset was 11 076 bp and the total aligned length for ITS was 733 bp including only a few small (tri- or tetranucleotide) insertions. The aligned concatenated dataset of ITS and the 7 cpDNA loci was 11 809 bp (Table 2).

**Table 2:** Summary of the cpDNA loci and the nDNA locus ITS used in this study

							Average Pairwise Difference (range)^2^		
								
Locus	N	Aligned Length (bp)	Characters Constant (percent)	Parsimony Informative Characters (percent)	Autapo-morphies	Nucleotide Substitution Model^1^	Within Species	Among Species	Primer Source
							n/a	3.12%	see (Neel and Cummings 2004)
			(88.40%)	(3.68%)				(0 – 6.20%)	
*ndh*F	6	2131	2002	66	63	TVM	n/a	2.97%	see (Neel and Cummings 2004)
			(93.95%)	(3.10%)				(0.42 – 5.00%)	
*rbc*L	37	1331	1205	53	73	GTR+I	0.17%	1.07%	see (Neel and Cummings 2004)
			(90.53%)	(4.00%)			(0–0.39%)	(0 – 3.07%)	
*rpo*B	78	375	306	52	17	GTR+Γ	0.31%	2.15%	http://www.kew.org/barcoding/update.html
			(81.60%)	(13.87%)			(0–3.19%)	(0 – 5.40%)	
*rps*2	77	665	520	135	10	TVM+Γ+I	0.11%	4.37%	de Pamphilis et al. 1997
			(78.20%)	(20.30%)			(0–2.84%)	(0 – 8.59%)	
*trn*T-*trn*F	79	1868	1479	320	69	TVM+Γ+I	0.29%	3.24%	Taberlet et al. 1991
			(79.68%)	(17.13%)			(0–3.29%)	(0 – 6.04%)	
*psb*A-*trn*H	79	884	669	189	26	TVM+Γ	0.20%	7.75%	Sang et al. 1997; Tate and Simpson 2003
			(75.68%)	(21.38%)			(0–2.94%)	(0 – 20.50%)	
All cpDNA Loci	79	11076	9592	950	545	TVM+Γ+I	0.31%	3.82%	
			(86.51%)	(8.57%)			(0–2.0%)	(0 – 7.40%)	
ITS	68	733	504	175	54	GTR+Γ+I	0.75%	6.51%	White et al. 1990
			(68.76%)	(23.87%)			(0–3.93%)	(0.14 – 21.26%)	
All Loci	79	11809	10096	1125	599	GTR+Γ+I	0.36%	4.05%	
			(85.41%)	(9.52%)			(0.02–1.94%)	(0.04–7.99%)	

N = the number of *Agalinis *species for each locus. Pairwise distances were calculated using *Agalinis *species only and do not include outgroup taxa.

^1 ^Nucleotide substitution model as selected using MODELTEST.

^2 ^Pairwise differences are based on the maximum likelihood distances calculated using the nucleotide substitution parameters associated with the best fitting model identified using MODELTEST

The percent of constant characters among *Agalinis *species varied from 68.76% – 93.95% for ITS and *ndh*F respectively (Table 2). After *ndh*F, *rbc*L had the largest percentage of constant characters (90.53%). The number of parsimony informative sites for individual loci ranged from 52 (*rpo*B) to 320 (*trn*T-*trn*F) (Table 2). ITS exhibited the widest range of pair-wise maximum likelihood distances among species within the genus, ranging from 0.14% – 21.26%. The most conserved locus was *rbc*L with pairwise distances among *Agalinis *species ranging from 0 – 3.07% and averaging 1.07%; *psb*A-*trn*H had the largest range of among-species pairwise maximum likelihood distance of all the cpDNA loci, ranging from 0 – 20.50% and averaging 7.75% (Table 2).

Levels of variation we observed were similar to those found in other phylogenetic studies of congeneric species. The extensive length variation we observed in *trn*T-*trn*F (shortest sequence length of 1228 bp compared to the length of the alignment of 1868 bp) has also been observed within the confamilial genus *Pedicularis *[75]. A study of *Mimulus *(Phrymaceae) [2] in which only the *trn*L-*trn*F portion of *trn*T-*trn*F was sampled found a similar degree of variability expressed as the percent of parsimony informative characters (20.7% compared to 17.13% observed in this study). In *Lymania *(Bromeliaceae) 577 of 602 (96%) bases of *psb*A-*trn*H were constant [76] compared to 669 of 884 (75.68%) constant characters within this study. The maximum level of variation we observed at the nuclear ITS locus (ML distance = 21.26%) is similar to that found in other genera within the Orobanchaceae (*Pedicularis *[77] and *Orobanche *[78]).

### General phylogenetic hypotheses

**Figure 1:**
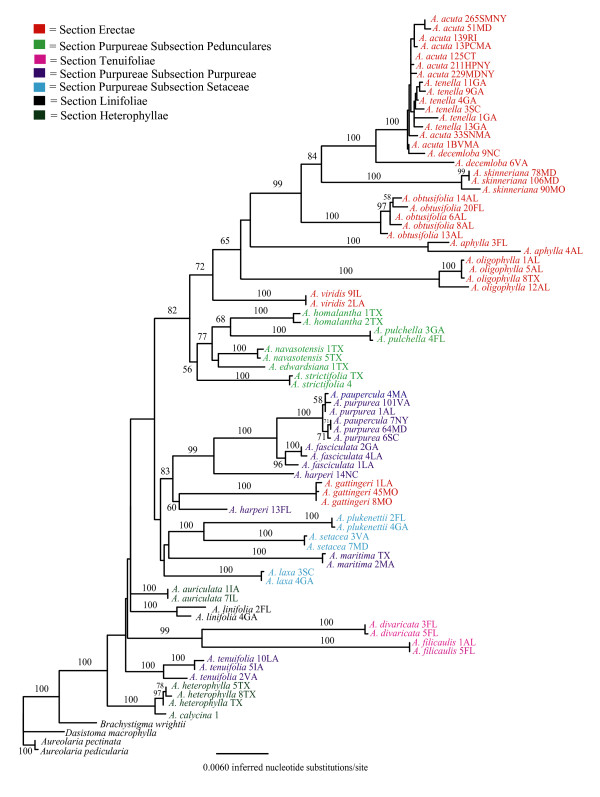
**Phylogenetic tree depicting evolutionary relationships among sampled taxa based on seven cpDNA loci. **Branch lengths depict the inferred number of nucleotide substitutions per site. Numerals at nodes represent the percent of 1000 bootstrap replicates supporting that clade. The ln likelihood of the tree is -30816.271.

**Figure 2:**
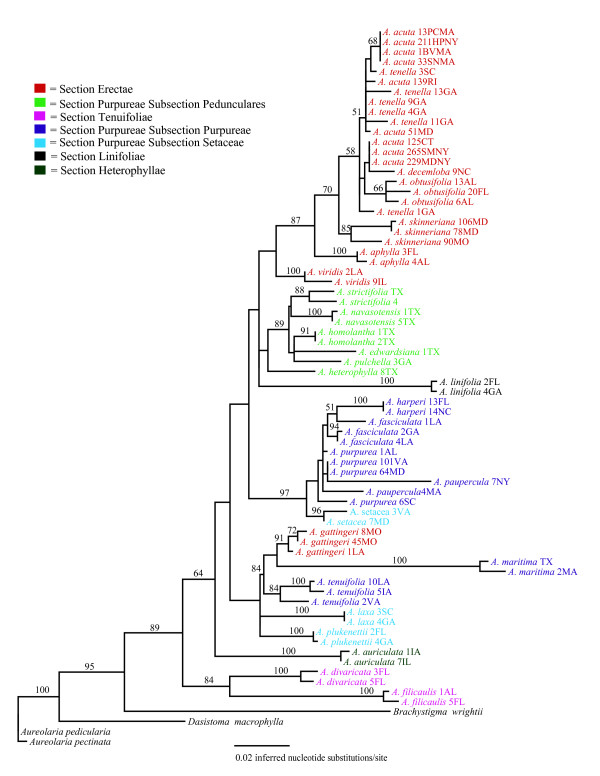
**Phylogenetic tree depicting evolutionary relationships among sampled taxa based on the nDNA ITS locus. **Branch lengths depict the inferred number of nucleotide substitutions per site. Numerals at nodes represent the percent of 1000 bootstrap replicates supporting that clade. The ln likelihood of the tree is -4250.1813.

**Figure 3:**
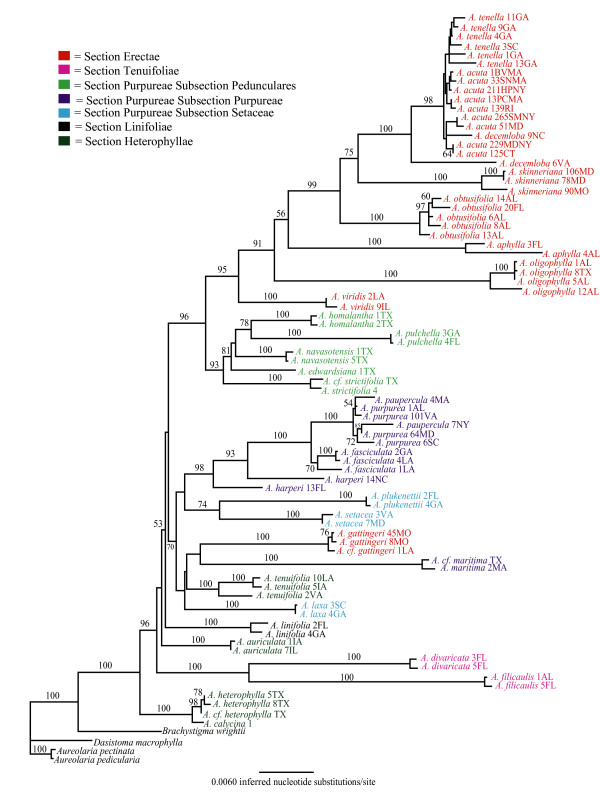
**Phylogenetic tree depicting evolutionary relationships among sampled taxa based on a concatenated dataset of the seven cpDNA loci and the nDNA ITS locus.** Branch lengths depict the inferred number of nucleotide substitutions per site. Numerals at nodes represent the percent of 1000 bootstrap replicates supporting that clade. The ln likelihood of the tree is -35900.524.

The phylogenies inferred from the three data matrices differed in tree shape and support for specific relationships (Figs 1, 2, 3). Results of the AU test [73] suggested that topologies derived from the cpDNA and the complete data set (Figs. 1 &3) were significantly different from one another (*P *< 0.05). To rule out the possibility that samples missing from the ITS dataset (Additional file 1) were causing some of the incongruence with the cpDNA phylogeny a reduced data matrix of the cpDNA loci was created that included only those samples also present in the ITS dataset. Results of the AU test (*P *< 0.05), indicated that the resulting topology (data not shown) was similar to the one from the complete cpDNA dataset, suggesting that missing individuals are not responsible for the incongruence between the nuclear and chloroplast DNA datasets.

Incongruences between phylogenies based on nDNA and cpDNA are not uncommon [e.g., [79-81]] and can indicate specific biological processes in species evolution. For example, hybridization has often been posited as an explanation for incongruence [82,83]. Alternatively, differences between the topologies might simply reflect the stochastic nature of the coalescent process [84]. The lack of bootstrap support for many of the internal nodes on the phylogenies (particularly those on the phylogeny inferred with the ITS dataset) prevent us from making strong statements regarding the meaning of the incongruences. Our discussion of relationships among putative taxa relies primarily on the full and chloroplast data sets because they tended to provide better support for inferred relationships. We point out specific instances where the estimates of relationships are different and well supported in the ITS data set.

All three topologies we examined provided strong statistical support for the monophyly of the sampled *Agalinis *species relative to the sampled outgroup species. Species now recognized as *Agalinis *have variously been included in the genera *Gerardia, Tomanthera*, and *Virgularia*. *Gerardia *had previously been applied to another taxon and the name was abandoned in favor of *Agalinis *[31]; *Tomanthera *and *Virgularia *are now synonymous with *Agalinis. Aureolaria*, *Brachystigma*, *Dasistoma*, *Seymeria*, and *Esterhazya *are considered close allies and at times have been considered congeneric with *Agalinis *[22,23]. Morphological evidence suggested *Agalinis *was a distinct genus from *Brachystigma *and *Aureolaria *[33], which our results clearly support. Phylogenetic analysis of the Orobanchaceae based on a single locus (phytochrome A) [85] indicates that the South American genus *Esterhazya *may be more closely related to *Agalinis *than are *Aureolaria *or *Seymeria*. South American species of *Agalinis *have not been included in any systematic studies and the only publicly available sequence from *Esterhazya *represents a locus we did not sample [87]. Sampling additional *Esterhazya *species and South American *Agalinis *species will be essential to fully understanding evolutionary relationships in this group as a whole and to confirm the monophyly of the genus.

### Section-level hypotheses

*Agalinis linifolia *is the only perennial *Agalinis *species in North America and has additional distinguishing characters that have resulted in placement in its own monotypic section (Table 1) that has been suggested to be basal to the rest of the species. The unique ensemble of traits includes the type of thickenings on the inner walls of the seed coat cells [34], lack of yellow lines on the corolla, dense pubescence at the bases of the posterior corolla lobes, presence of aerenchyma in stems, conspicuous endodermis in roots, and palisade tissue in leaves that is developed more strongly towards the lower surface [23]. Monophyly of *A. linifolia *was supported; however, its placement within the genus remains ambiguous due to lack of support for surrounding nodes in all trees (Figs. 1, 2, 3). Despite this ambiguity, there is no evidence that this taxon is basal because it is placed within a relatively derived clade that is well supported. Further, a tree placing *A. linifolia *as basal had a significantly worse likelihood score than the best tree from the unconstrained analyses (P < 0.05). Thus, the perennial growth habit appears to be derived within this genus of otherwise annual species.

Members of Section Heterophyllae have also been suggested to be basal within the genus based on having relatively large, broad, lanceolate leaves; leaf-like calyx lobes that are longer than the calyx tube; oblong or ovoid-oblong capsules, and glabrous stems [22]. In particular, Pennell [22] suggested that *A. auriculata *most closely resembled the ancestral state of the genus based on also having relatively large corollas with pubescence limited to the area below the posterior sinus, and having posterior anther cells that are smaller than the anterior cells in addition to the characters described above [23]. Diagnostic aspects of leaf anatomy include thickened epidermal cell walls, bands of sclerids and fibers between the cortex and phloem, patterns of the subepidermal collenchyma on the leaf midribs, and lack of specialized trichomes [42,43]. We found only two of the three species hypothesized to comprise this section (*A. heterophylla *and *A. calycina*) to be monophyletic, and this well supported clade is indeed basal within the genus in the cpDNA and full data sets. Of these two species, we could obtain ITS sequence only for *A. heterophylla*, which was placed sister to the Subsection Pedunculares clade, but with low bootstrap support. The third species, *A. auriculata*, is not closely related to this group in any of the trees (Figs. 1, 2, 3). *Agalinis auriculata *was known to differ from *A. heterophylla *in leaf and stem pubescence [42], and the perceived importance of differences suggested by those features are supported by our molecular data (Figs. 1, 2, 3).

Section Tenuifolieae has long been taxonomically problematic [42]. Pennell [23] united *Agalinis tenuifolia*, *A. divaricata *and *A. filicaulis *in this section based on lack of pubescence on the posterior corolla and upper corolla lobes being arched forward rather than erect or reflexed back as is seen in the rest of the genus. Canne-Hilliker and Kampny [42] placed *A. tenuifolia *in Section Purpureae based on morphological and anatomical features, while retaining *A. divaricata *and *A. filicaulis *in Section Tenuifolieae. Most obviously, the upper corolla lobes in *A. divaricata *and *A. filicaulis *are less than 1/3 the length of the lower lobes and the corolla is greatly flattened, occluding the opening to the throat. In contrast, the upper corolla lobes of *A. tenuifolia *are more equal in length to the lower lobes and the corolla throat is closer to round in cross section. *Agalinis divaricata *and *A. filicaulis *also share peculiar seedling and trichome types [41] and stem anatomy [42] that are not similar to any other *Agalinis *species and thus their placement has been challenging. High bootstrap support and the relatively long branch length supporting this clade in both the cpDNA and nDNA trees (Figs. 1, 2, 3) strongly support a sister relationship between *A. divaricata *and *A. filicaulis*. At the same time, branch lengths separating these two species are the longest of any sister-taxon pairs in the data set (Fig. 3). Relationships of this clade to other members of the genus depicted in the cpDNA tree conflict with those in the ITS tree. The cpDNA sequence data indicate that the most likely placement of the *A. divaricata*/*A. filicaulis *clade is sister to a clade including Section Purpureae (Fig. 1), and in the phylogenies from the ITS and the full data set these species have a more basal placement within the genus (Figs. 2 &3).

Relationships of *A. tenuifolia *to other taxa are ambiguous; the cpDNA phylogeny supports a sister relationship of *A. tenuifolia *with all *Agalinis *species except the *A. heterophylla/A. calycina *clade (bootstrap support = 100%) (Fig. 1). Phylogenies based on the ITS and full data sets indicate an alliance with *A. maritima *and *A. gattingeri *(bootstrap support = 84%) (Figs. 2 &3). In no case, however, does this species appear to be closely related to *A. divaricata *and *A. filicaulis*.

With the exception of *A. gattingeri*, which is found within the clade discussed above, the monophyly of Section Erectae is strongly supported in the full data tree (bootstrap support = 95%) (Fig. 3). This section is united by the following genetic, anatomical, and morphological characters: chromosome number of *n *= 13 [43], yellow-green colored foliage that does not blacken upon drying, small flowers that have relatively short corolla tubes and reflexed corolla lobes, pedicels longer than the calyx tube and light brown seeds [34,42]. Lack of blackening upon drying is thought to be due to low concentrations of aucubosides [38] that are at higher concentrations in other members of the genus. Placement of *A. gattingeri *apart from other members of the Erectae is problematic because it contradicts evidence that suggests close evolutionary relationships based on chromosome number [45] and the unique seed type [34] shared by other members of the section. However, *A. gattingeri *was always considered peripheral within Section Erectae due to its lack of anatomical features of the stem that are characteristic of the rest of the group [43]. Additional sampling is necessary to determine if this placement outside the Erectae is accurate or due to misidentification of the collections we sequenced or misinterpretation of the anatomical and morphological features.

Section Purpureae as defined by both Canne-Hilliker and Pennell was the largest section in the genus and it has been considered to have 3–5 subsections. Members were united by having globose capsules, dark brown seeds, narrow leaves that turn black upon drying and calyx lobes that are shorter than the calyx tube. We found little support for any of the historical concepts of this section or the majority of the recognized subsections (Figs. 1, 2, 3). Only Subsection Pedunculares appears to be a natural group (Figs. 1 &3); however, this subsection is sister to taxa comprising Section Erectae rather than to other taxa considered to be in the Purpureae. Subsection Pedunculares was considered to be distinct from the Erectae based on corolla form and pubescence patterns, seed color and surface patterns, and stem and leaf anatomy [43]. Neel and Cummings [47] had previously suggested a sister relationship between the Pedunculares and the Erectae but their results were based on fewer species and the relationship did not have strong bootstrap support. Aligning the Pedunculares with the Erectae unites all the taxa with 13 chromosomes except *A. gattingeri*, which is placed with species considered to be in Section Purpureae. If not for the problematic placement of *A. gattingeri*, it would appear that *n *= 14 was ancestral and the haploid chromosome number of 13 arose only once in the genus.

We found no support for the monophyly of Subsections Purpureae or Setaceae and many nodes supporting members that have been recognized to comprise these groups have weak bootstrap support (Figs. 1, 2, 3). Thus, despite the fact that most species are separated by well supported branches with non-zero lengths (exceptions will be discussed below), higher level relationships among species remain unclear. We did, however, find support for some hypothesized relationships. For example, our data support close relationships of *A. fasciculata*, *A. purpurea*, and *A. paupercula *in all trees (Figs 1, 2, 3). We also found support for a sister relationship between *A. setacea *and *A. plukenettii *in the full data set (Fig. 3) that had previously been hypothesized based on both species having acute trichomes [41]. We found no support for a close relationship of *A. laxa *to these two taxa in the full data set but *A. laxa *and *A. plukenettii *were part of a poorly supported clade that also comprised the *A. tenuifolia*/*A. gattingeri*/*A. maritima *clade in the ITS tree. Although it had been classified with *A. setacea *and *A. plukenettii *in Subsection Setaceae, *Agalinis laxa *was known to differ in having capitate trichomes on the hypocotyls and lacking acute trichomes [41].

Overall, our results suggest that North American members of the genus comprise six major lineages, however we were not able to resolve branching order among many of these lineages. We propose that Section Heterophyllae consisting of *A. calycina*, *A. heterophylla *(and potentially *A. densiflora *but we did not sample this species) represents the basal group. Following the divergence of Section Heterophyllae, a rapid diversification resulted in five additional primary lineages. These lineages include one comprising what have been considered Section Erectae and Subsection Pedunculares, two unrelated monospecific lineages (one comprising *A. auriculata *and the other *A. linifolia*), a fourth lineage corresponding roughly to Section Tenuifolieae, and a fifth consisting of the remaining taxa that have been included in Section Purpureae. We further recognize Section Erectae (sans *A. gattingeri*) and what was Subsection Pedunculares as distinct sister lineages that are relatively derived within the genus.

One potential explanation for lack of bootstrap support for the more basal relationships in the genus is a rapid diversification of lineages (i.e., a hard polytomy) [e.g., [86]]. This explanation is also supported by presence of comparatively short branches towards the base of the phylogeny (Fig. 3). Alternatively our data may simply not be sufficient to determine the order of branching (i.e., a soft polytomy) [e.g., [86]]. Although there is the potential that sequencing additional nuclear loci may be able to resolve the branching order at interior nodes on the phylogeny, we believe that a soft polytomy seems unlikely given the amount of DNA sequence we sampled and the levels of variation we observed in those sequences. Because our objectives included estimating both deep and shallow relationships, we specifically chose an array of loci that were expected to be useful for estimating relationships across the ranges of divergence anticipated.

### Testing Species-level hypotheses

Our results corroborate most of the species designations in the genus and clarify some previous taxonomic ambiguities. Based on likelihood ratio tests, 83% (24 of 29) sampled species in the genus have significant (non-zero) branch lengths and 78% of species with multiple samples have bootstrap support > 98%. There are also multiple cases in which branch lengths between conspecific individuals are greater than zero (e.g. the two *A. aphylla *samples [Fig. 3]) indicating that there is substantial differentiation among conspecific populations. Although we do not believe that there is a single particular amount of differentiation that determines a cutoff for recognizing a species-level distinction, we do expect populations within species to lack strong hierarchical structure due to tokogenetic processes [87]. The hierarchical structure indicated by significant branch lengths and high bootstrap support within species (e.g., *A. skinneriana*, *A. decemloba*, *A. oligophylla*, *A. fasciculata*, and *A. tenuifolia*) indicates the need for closer examination of the biological basis of the observed patterns. Sampling additional loci and populations and using phylogeographic analytical methods would contribute to understanding whether these populations actually represent different species.

Exceptions to overall pattern of monophyly described above are the apparent polyphyly of *A. harperi*; the lack of differentiation between *A. purpurea *and *A. paupercula*; and the lack of differentiation among *A. decemloba, A. tenella*, and *A. acuta *(Figs. 1, 2, 3). All rare species that are of conservation concern except *A. acuta *and *A. paupercula *were supported as distinct.

The two sampled *A. harperi *individuals had identical ITS sequences (Fig. 2) but were polyphyletic based on cpDNA data (Fig. 1). Both individuals were part of a moderately supported clade (bootstrap support = 83%) consisting of representatives of putative *A. purpurea *and *A. paupercula*, *A. fasciculata *and *A. gattingeri *individuals. However, *A. harperi *13FL is most closely related to *A. gattingeri *and the other appears sister to the *A. fasciculata*/*A. purpurea/A. paupercula *clade (Fig. 1). Reamplification and sequencing of *rpo*B, *rps*2, and *trn*T-*trn*F loci from the two *A. harperi *samples yielded sequences that were identical to those used in constructing the phylogenies, thus ruling out the possibility that samples were mishandled. Therefore, the difference between the cpDNA and nDNA may be best explained by hybridization or introgression from another species that is represented by chloroplast capture [88]. Although it is not possible to say with much certainty given the lack of statistical support for the relationship between the *A gattingeri *samples and *A. harperi *13FL, *A. gattingeri *may be the species from which cpDNA has introgressed into the *A. harperi *collection from Florida. Sampling from more *A. harperi *and *A. gattingeri *individuals and populations is required to resolve this issue.

*Agalinis purpurea *and *A. paupercula *have been the subject of debate, with taxonomic hypotheses ranging from treating them as two species, as two varieties of *A. purpurea*, or synonymizing them under a single species. Pennell suggested relatively recent divergence related to the last ice age [22]. These putative taxa differ from one another in that *A. paupercula *is reported to have smaller corollas (10–20 mm) and broader calyx lobes that are greater than half the length of the calyx tube [22]. *Agalinis purpurea *has corollas ranging from 18–38 mm long and narrow calyx lobes that are less than half the length of the tube. Although they share many features during floral ontogeny, they do differ in *A. paupercula *var. *borealis *having different anther orientation, filament insertion points closer to the ovary height, later stigma initiation, and less exsertion of the stigma at anthesis than *A. purpurea *[44]. The effect of these characteristics on mating system or reproductive isolation is unknown. It is also not known if these characteristics extend to other *A. paupercula *varieties. Our data do not support recognizing *A. paupercula *as a distinct taxon. However, we did not thoroughly sample from a large number of putative populations of *A. paupercula *and it remains possible that some populations that have been attributed to that species represent a distinct entity. Further it is possible that higher resolution markers would allow us to differentiate *A. paupercula *and *A. purpurea *as is discussed below for *A. acuta*.

One of the primary objectives of this study was to evaluate the evolutionary distinctiveness of the federally listed endangered species *Agalinis acuta*. Potential synonymy of *A. acuta *with *A. tenella *was raised by Neel and Cummings [47] due to lack of sequence divergence in two cpDNA loci between two individuals. Sampling nine representatives of *A. acuta *and five of *A. tenella *in this study allowed us to more thoroughly examine this issue. Previous taxonomic revisions [53] that synonymized *A. tenella *and *A. decemloba *with *A. obtusifolia *necessitated inclusion of accessions attributed to the latter two species. Rather than clarifying relationships among these taxa, our results show a more convoluted situation than was previously thought to exist. The ITS phylogeny shows *A. tenella*, *A. acuta*, *A. decemloba *and *A. obtusifolia *to be polyphyletic (Fig. 3); however, there is little support for this topology. The phylogenies based on cpDNA loci alone and all loci combined (Figs. 1 &3, respectively) show *A. tenella *to be monophyletic and subtended by a branch with a length that is significantly different from zero based on the likelihood ratio test. There is, however, no bootstrap support for this clade and it is nested within a clade that includes *A. acuta *and *A. decemloba*. On both topologies, *A. acuta *and *A. decemloba *are polyphyletic (Figs. 1 &3) and an AU test indicated that forcing the monophyly of *A. acuta *and *A. tenella *yielded a topology that was significantly worse than the best topology for all three datasets (*P *< 0.05). Although *A. acuta, A. decemloba*, and *A. tenella *form a highly supported monophyletic clade, one accession of *A. decemloba *(6VA) is distinguished from all other accessions of these three taxa based on branch lengths that are significantly different than zero and 98% bootstrap support (Fig. 3). This differentiation is the result of differences within the *trn*T-*trn*F locus. These include numerous single nucleotide differences and a 16 bp deletion, the majority of which are also present in the *A. obtusifolia *samples.

Regardless, our results do not provide statistical support for separate species status for *A. acuta*, *A. decemloba*, and *A. tenella *under the criteria of either a phylogenetic species concept [89] or a genealogical species concept [11]. *Agalinis obtusifolia *comprises a monophyletic clade that is sister to the clade containing *A. skinneriana*, and *A. tenella*, *A. acuta*, and *A. decemloba*, thus strongly refuting the recent taxonomic revision synonymizing both *A. decemloba *and *A. tenella *with *A. obtusifolia *[e.g., [27,53]].

Lack of monophyly of even morphologically well defined species can result from incomplete lineage sorting of shared ancestral polymorphism or contemporary gene flow [16,17,90]. Given that it takes on the order of ~8.7 N_e _generations for an 0.95 probability of reciprocal monophyly to evolve at a single locus after speciation events [17,20], it can be challenging to distinguish among closely related taxa using phylogenetic methods. It is also possible that the DNA sequences we examined do not have sufficient mutation rates to have accumulated nucleotide differences in the time since divergence. However, the loci sampled appear to have a sufficient amount of variation to distinguish ~83% of the 29 sampled species in the genus, some of which are likely to have recently diverged from a common ancestor. Due to the important policy implications of combining *A. acuta*, *A. tenella*, and *A. decemloba *into a single taxon, additional research is being conducted on the morphological and genetic differences, using more variable loci, from samples collected from throughout the range of each species.

As mentioned above, we found that *A. skinneriana *formed a well supported clade that was sister to the clade containing *A. tenella*, *A. decemloba*, and *A. acuta *(Figs. 1 &3). Prior to this work, taxonomic boundaries and phylogenetic affinities of *A. skinneriana *were not understood. Additionally, the Maryland populations that we sampled were problematic for experts to identify because these populations were beyond the known range for the species at the time they were discovered. The morphological characteristics of *A. skinneriana *most closely matched these populations, but there was some lingering question as to their identity. Our results confirm that these populations are sister to the *A. skinneriana *sample from Missouri and they represent an extension of this otherwise Midwestern prairie taxon to the grasslands of the Atlantic coastal plain. However, the branch separating the Maryland populations from the Missouri population is significantly different from zero indicating that further investigation of the phylogeography of this putative species may be warranted to determine if the Maryland populations are actually an unrecognized species. Clarifying these relationships is important because this species is considered rare in the state of Maryland and correct identification is essential for both protecting a rare entity and not imposing restrictions for something that does not warrant them.

## Conclusion

In conclusion, the sampled *Agalinis *species form a well supported, monophyletic group relative to the other genera sampled from within the family Orobanchaceae. Despite the well known taxonomic difficulty in this genus, 24 of the 29 the species we sampled that had been recognized based on anatomy and morphology were well supported. We confirmed the monophyly of 19 rare species, thus supporting their eligibility for receiving conservation attention. The species that do not form well supported clades based on DNA sequence data include the federally listed species *A. acuta *and the state-rare species *A. paupercula*. Although we were able to resolve some relationships among these species, most notably that the synonymization of the latter two with *A. obtusifolia *is unwarranted, a number of ambiguities remain. Due to the important policy implications raised by this finding, we are examining relationships among *A. acuta, A. decemloba*, and *A. tenella *further by sampling more individuals and populations using higher resolution molecular markers and morphological data. It is clear that most hypotheses regarding section- and subsection-level relationships based on morphology are not supported and taxonomic revisions are warranted.

## Authors' contributions

MCN conceived of the study and obtained funding. JBP carried out laboratory work and analyses. Both authors participated in sequence alignment, interpretation of results, and writing of the manuscript.

## Supplementary Material

Additional file 1 **General population locations and Genbank accession numbers for loci sampled from North American *Agalinis *species examined in this study.** Section and subsection classifications follow J.M. Canne-Hilliker. Genbank accession numbers for those sequences with "N & C (2004)" can be found in Neel and Cummings (2004). Click here for file
