# Supplementary material for: Phylogenetic patterns and conservation among North American members of the genus Agalinis (Orobanchaceae)
Source: BMC Evol Biol. 2008 Sep 26;8:264. doi: 10.1186/1471-2148-8-264 (PMC2564944; doi:10.1186/1471-2148-8-264)
Supplement: Additional file 1 — General population locations and Genbank accession numbers for loci sampled from North American Agalinis species examined in this study. Section and subsection classifications follow J.M. Canne-Hilliker. Genbank accession numbers for those sequences with "N & C (2004)" can be found in Neel and Cummings (2004). [file 1471-2148-8-264-S1.pdf]

Additional file 1. General population locations and Genbank accession numbers for loci sampled from North American *Agalinis* species examined in this study. Section and subsection classifications follow J.M. Canne-Hilliker. Genbank accession numbers for those sequences with “N & C (2004)” can be found in Neel and Cummings (2004).

**Taxon**	**Sampled Location**	***mat*K**	***rbc*L**	***ndh*F**	***rps*2**	***trn*T -*trn*F**	***trn*H-*psb*A**	***rpo*B**	**ITS**
**Section Erectae**									
A. acuta 125CT	Windham Co., CT				EU827965	EU828128	EU827882	EU828046	EU827810
A. acuta 139RI	Washington Co., RI				EU827966	EU828129	EU827883	EU828047	EU827811
A. acuta 13PCMA	Sandwich, Barnstable Co. MA				EU827967	EU828130	EU827884	EU828048	EU827812
A. acuta 1BVMA	Waquoit Bay, Barnstable Co. MA	N & C (2004)	N & C (2004)	N & C (2004)	EU827968	EU828131	EU827885	EU828049	EU827813
A. acuta 211HPNY	Nassau Co., NY				EU827969	EU828132	EU827886	EU828050	EU827814
A. acuta 229MDNY	Montauk Downs, Suffolk Co. NY				EU827970	EU828133	EU827887	EU828051	EU827815
A. acuta 265SMNY	Shadmoor,Suffolk Co., NY				EU827971	EU828134	EU827888	EU828052	EU827816
A. acuta 33SNMA	Dukes Co., MA				EU827972	EU828135	EU827889	EU828053	EU827817
A. acuta 51MD	Baltimore Co., MD				EU827973	EU828136	EU827890	EU828054	EU827818
A. aphylla 3FL	Liberty Co., FL	N & C (2004)	N & C (2004)	N & C (2004)	EU827974	EU828137	EU827891	EU828055	EU827819
A. aphylla 4AL	Geneva Co., AL				EU827975	EU828138	EU827892	EU828056	EU827820
A. decemloba 6VA	Lunenberg Co., VA	EU828211	EU828220		EU827982	EU828145	EU827899	EU828063	
A. decemloba 9NC	Randolph Co., NC				EU827983	EU828146	EU827900	EU828064	EU827826
A. gattingeri 45MO	Crawford Co., MO		EU828224		EU827993	EU828156	EU827910	EU828074	EU827836
A. gattingeri 8MO	Hickory Co., MO				EU827994	EU828157	EU827911	EU828075	EU827835
A. gattingeri 1LA	Webster Parish, LA				EU827992	EU828155	EU827909	EU828073	EU827823
A. obtusifolia 13AL	Geneva Co., AL				EU828008	EU828171	EU827925	EU828089	EU827849
A. obtusifolia 14AL	Mobile Co., AL		EU828233			EU828172	EU827926	EU828089	EU827851
A. obtusifolia 20FL	Liberty Co., FL	N & C (2004)	N & C (2004)	N & C (2004)	EU828010	EU828174	EU827928	EU828092	EU827852
A. obtusifolia 6AL	Mobile Co., AL				EU828011	EU828175	EU827929	EU828093	
A. obtusifolia 8AL	Geneva Co., AL		EU828234			EU828176	EU827930	EU828094	
A. oligophylla 12AL	Tyler Co., TX				EU828012	EU828177	EU827931	EU828095	
A. oligophylla 1AL	Mobile Co., AL	EU828216	EU828235		EU828013	EU828178	EU827932	EU828096	
A. oligophylla 5AL	Mobile Co., AL				EU828014	EU828179	EU827933	EU828097	
A. oligophylla 8TX	Vernon Parish, LA				EU828015	EU828180	EU827934	EU828098	

A. skinneriana 106MD	Prince Georges Co., MD		EU828239		EU828028	EU828193	EU827947	EU828110	EU827864
A. skinneriana 78MD	Dorchester Co., MD				EU828029	EU828194	EU827948	EU828111	EU827865
A. skinneriana 90MO	Vernon Co., MO		EU828240		EU828030	EU828195	EU827949	EU828112	EU827866
A. tenella 1GA	Ware Co., GA	EU828215	EU828241		EU828009	EU828173	EU827927	EU828091	EU827850
A. tenella 11GA	Lowndes Co., GA				EU828032	EU828197	EU827951	EU828114	EU827868
A. tenella 13GA	Grady Co., GA				EU828033	EU828198	EU827952	EU828115	EU827869
A. tenella 3SC	Colleton Co., SC				EU828034	EU828199	EU827953	EU828116	EU827870
A. tenella 4GA	Ware Co., GA	N & C (2004)	N & C (2004)		EU828035	EU828200	EU827954	EU828117	EU827871
A. tenella 9GA	Lanier Co., GA				EU828036	EU828201	EU827955	EU828118	EU827872
A. viridis 2LA	Natchitoches Parish, LA	EU828218	EU828242		EU828040	EU828205	EU827959	EU828122	EU827876
A. viridis 9IL	DeSoto Parish, LA				EU828041	EU828206	EU827960	EU828123	EU827877
**Section Heterophyllae**									
A. auriculata 1IA	Story Co., IA				EU827976	EU828139	EU827893	EU828057	EU827821
A. auriculata 7IL	Will Co., IL	N & C (2004)	N & C (2004)		EU827977	EU828140	EU827894	EU828058	EU827822
A. calycina	Pecos Co., TX		EU828219		EU827978	EU828141	EU827895	EU828059	
A. heterophylla 5TX	Cameron Co., TX				EU827997	EU828160	EU827914	EU828078	
A. heterophylla 8TX	Stephens Co., TX				EU827998	EU828161	EU827915	EU828079	EU827839
A. heterophylla TX	Grimes Co., TX	N & C (2004)	N & C (2004)	N & C (2004)	EU827979	EU828142	EU827896	EU828060	
**Section Linifoliae**									
A. linifolia 2FL	Liberty Co., FL	N & C (2004)	N & C (2004)	N & C (2004)	EU828003	EU828166	EU827920	EU828084	EU827844
A. linifolia 4GA	Cinch Co., GA		EU828231		EU828004	EU828167	EU827921	EU828085	EU827845
**Section Purpureae**									
Subsection Pedunculares									
A. edwardsiana 1TX	Stephens Co., TX	EU828212	EU828221		EU827986	EU828149	EU827903	EU828067	EU827829
A. homalantha 1TX	Tyler Co., TX		EU828227		EU827999	EU828162	EU827916	EU828080	EU827840
A. homalantha 2TX	Jasper Co., TX		EU828228		EU828000	EU828163	EU827917	EU828081	EU827841
A. pulchella 3GA	Grady Co., GA	N & C (2004)	N & C (2004)		EU828020	EU828185	EU827939	EU828102	EU827857
A. pulchella 4FL	Florida		EU828237		EU828021	EU828186	EU827940	EU828103	
A. strictifolia 4	Stephens Co., TX				EU828031	EU828196	EU827950	EU828113	EU827867
A. strictifolia TX	Cameron Co., TX				EU827981	EU828144	EU827898	EU828062	EU827825
A. navasotensis 1TX	Tyler Co. TX		EU828232		EU828006	EU828169	EU827923	EU828087	EU827847

A. navasotensis 5TX	Grimes Co., TX				EU828007	EU828170	EU827924	EU828088	EU827848
Subsection Purpureae									
A. fasciculata 1LA	Grimes Co., TX	EU828213	EU828222		EU827987	EU828150	EU827904	EU828068	EU827830
A. fasciculata 2GA	Long Co., GA	N & C (2004)	N & C (2004)		EU827988	EU828151	EU827905	EU828069	EU827831
A. fasciculata 4LA	Caddo Parish, LA				EU827989	EU828152	EU827906	EU828070	EU827832
A. harperi 13FL	Liberty Co., FL		EU828225		EU827995	EU828158	EU827912	EU828076	EU827837
A. harperi 14NC	Brunswick Co., NC		EU828226		EU827996	EU828159	EU827913	EU828077	EU827838
A.. maritima TX	Cameron Co., Texas				EU827980	EU828143	EU827897	EU828061	EU827824
A. maritima 2MA	Barnstable CO. MA				EU828005	EU828168	EU827922	EU828086	EU827846
A. paupercula 4MA	Barnstable CO. MA				EU828016	EU828181	EU827935	EU828099	EU827853
A. paupercula 7NY	Shadmoor, Suffolk Co. NY				EU828017	EU828182	EU827936	EU828100	EU827854
A. purpurea 101VA	Fauquier Co, VA				EU828022	EU828187	EU827941	EU828104	EU827858
A. purpurea 1AL	Mobile Co., AL	EU828217	EU828238		EU828023	EU828188	EU827942	EU828105	EU827859
A. purpurea 64MD	Dorchester Co., MD				EU828024	EU828189	EU827943	EU828106	EU827860
A. purpurea 6SC	Harry Co. SC				EU828025	EU828190	EU8279414	EU828107	EU827861
A. tenuifolia 2VA	Prince Edward Co., VA				EU828038	EU828203	EU827957	EU828120	EU827874
A. tenuifolia 5IA	Story Co., Iowa	N & C (2004)	N & C (2004)	N & C (2004)	EU828039	EU828204	EU827958	EU828121	EU827875
A. tenuifolia 10LA	Caddo Parish, LA				EU828037	EU828202	EU827956	EU828119	EU827873
Subsection Setaceae									
A. laxa 3SC	Colleton Co., SC	EU828214	EU828229		EU828001	EU828164	EU827918	EU828082	EU827842
A. laxa 4GA	Long Co., GA		EU828230		EU828002	EU828165	EU827919	EU828083	EU827843
A. plukenettii 2FL	Washington Co., FL	N & C (2004)	N & C (2004)		EU828018	EU828183	EU827937		EU827855
A. plukenettii 4GA	Georgia		EU828236		EU828019	EU828184	EU827938	EU828101	EU827856
A. setacea 3VA	Prince Edward Co., VA	N & C (2004)	N & C (2004)		EU828026	EU828191	EU827945	EU828108	EU827862
A. setacea 7MD	Wicomico Co., MD				EU828027	EU828192	EU827946	EU828109	EU827863
**Section Tenuifolieae**									
A. filicaulis 5FL	Grady Co., GA		EU828223		EU827991	EU828154	EU827908	EU828072	EU827833
A. filicaulis 1AL	Mobile Co., AL				EU827990	EU828153	EU827907	EU828071	EU827834
A. divaricata 3FL	Liberty Co., FL				EU827985	EU828147	EU827901	EU828065	EU827827
A. divaricata 5FL	Washington Co., FL	N & C (2004)			EU827985	EU828148	EU827902	EU828066	EU827828
**Outgroup Species**									

Aureolaria pectinata	Liberty Co., Florida				EU828042	EU828206	EU827961	EU828124	EU827878
Aureolaria pedicularia	Prince Edward Co., VA	N & C (2004)	N & C (2004)	N & C (2004)	EU828043	EU828208	EU827962	EU828125	EU827879
Brachystigma wrightii	Cochise Co., AZ	N & C (2004)	N & C (2004)	N & C (2004)	EU828044	EU828209	EU827963	EU828126	EU827880
Dasistoma macrophylla	Ames, Iowa	N & C (2004)	N & C (2004)	N & C (2004)	EU828045	EU828210	EU827964	EU828127	EU827881
